# Review of functional and clinical relevance of intrinsic signal optical imaging in human brain mapping

**DOI:** 10.1117/1.NPh.4.3.031220

**Published:** 2017-06-09

**Authors:** Katherine A. Morone, Joseph S. Neimat, Anna W. Roe, Robert M. Friedman

**Affiliations:** a Vanderbilt University Medical Center, Department of Neurology, Nashville, Tennessee, United States; b University of Louisville School of Medicine, Department of Neurosurgery, Louisville, Kentucky, United States; c Oregon Health and Science University, Division of Neuroscience, Oregon National Primate Research Center, Beaverton, Oregon, United States; d Zhejiang University, Interdisciplinary Institute of Neuroscience and Technology, Qiushi Academy for Advanced Studies, HuaJiaChi Campus, Hangzhou, China

**Keywords:** human, operating room, intrinsic signal optical imaging, functional mapping

## Abstract

Intrinsic signal optical imaging (ISOI) within the first decade of its use in humans showed its capacity as a precise functional mapping tool. It is a powerful tool that can be used intraoperatively to help a surgeon to directly identify functional areas of the cerebral cortex. Its use is limited to the intraoperative setting as it requires a craniotomy and durotomy for direct visualization of the brain. It has been applied in humans to study language, somatosensory and visual cortices, cortical hemodynamics, epileptiform activity, and lesion delineation. Despite studies showing clear evidence of its usefulness in clinical care, its clinical use in humans has not grown. Impediments imposed by imaging in a human operating room setting have hindered such work. However, recent studies have been aimed at overcoming obstacles in clinical studies establishing the benefits of its use to patients. This review provides a description of ISOI and its use in human studies with an emphasis on the challenges that have hindered its widespread use and the recent studies that aim to overcome these hurdles. Clinical studies establishing the benefits of its use to patients would serve as the impetus for continued development and use in humans.

## 1 Introduction

Neurosurgical approaches are used to remove brain tumors and to resect tissue in diseases such as epilepsy. Common approaches for identifying diseased tissue include MRI scans and, in the case of epilepsy, electrical grid recordings prior to resection. However, these methods are still relatively crude and leave much room for improvement. This review discusses the use of intrinsic signal optical imaging (ISOI) in the human operating room (OR), its use as a functional brain mapping tool for research, and potential future trends for its practical intraoperative approach in the treatment of neurological disease. This review provides a brief description of ISOI, discusses the challenges of ISOI in the human OR, presents intriguing research findings in humans when applied to study language, somatosensory and visual cortices, cortical hemodynamics, epileptiform activity, and lesion delineation, and describes potential future directions for its practical intraoperative approach in the treatment of neurological disease.

### 1.1 Need for Greater Precision in Human Cortical Mapping

Precise information about structural and functional anatomy is essential in performing maximally effective and safe neurosurgical interventions. Functional brain mapping is necessary for identifying and preserving essential cortex during these interventions. A number of functional brain mapping methods have been adapted for patient care, including electrical stimulation mapping (ESM), electrophysiological recordings, and functional magnetic resonance imaging (fMRI). Other newer techniques are establishing their clinical role in functional brain mapping, such as functional near-infrared spectroscopy (fNIRS). Each has its own strengths and weaknesses. ESM is used to identify essential language cortex; however, it is unable to delineate these sites or to identify secondary language sites, and requires direct stimulation of cortex, raising concerns about triggering epileptic activity. Noninvasive electrophysiological recordings like electroencephalography (EEG) can be used to detect epileptic activity and with the recordings of sensory evoked potentials (EPs) allow for the identification of somatosensory and visual cortex. These methods offer high temporal resolution; however, intraoperatively they are recorded from grid electrodes with poor (typically 1 cm) spatial resolution. FMRI provides functional identification of targeted cortical areas. Importantly, it is noninvasive and can investigate the deep structures that ISOI cannot. As such, fMRI will continue to have an important role in preoperative planning and scientific research. However, alignment of preoperative images with intraoperative views is complicated by brain shift and swelling. fNIRS is a noninvasive optical brain imaging method that detects cortical activation, and though it has excellent temporal resolution (1 ms), it has poor (1 cm) spatial resolution. A functional mapping technique that offers higher spatial precision and is applied intraoperatively would provide more accurate information and lead to more informed intraoperative decision-making and greater patient benefit. ISOI has the advantage of potential intraoperative use allowing direct visualization of functional areas and improved surgical intervention.

ISOI is a functional imaging technique that uses a charge-coupled device (CCD) camera to indirectly measure neuronal activity by detecting hemodynamic changes related to neurovascular coupling. It requires craniotomy and durotomy, in other words, it requires direct exposure of the cortex to obtain its high spatial resolution functional maps of activity. It has been used extensively in animal research^1^^–^^5^ and shows promise for clinical applications.^6^ ISOI offers high spatial (∼100  μm) and temporal (100 ms) resolution and can be tuned to detect different physiological elements such as blood flow and oxygen consumption.^7^ This methodology has led to breakthroughs in our understanding of the functional organization, physiology, and pathophysiology of the brain.^7^ It has been used extensively in animals to study auditory,^8^^–^^10^ somatosensory,^11^^–^^13^ visual,^14^^–^^18^ and motor^19^ cortices in animals. The enormous advances in our understanding of cortical function resulting from ISOI studies led to concerted efforts to develop ISOI for clinical use.

### 1.2 Previous Intraoperative Studies

In 1992, using intraoperative ISOI, Haglund et al.^20^ first described their groundbreaking findings in human language cortex. This led to a surge of intraoperative ISOI studies in human language^21^^,^^22^ and somatosensory cortex (SI)^23^^–^^26^ as well as studies of cortical hemodynamics,^27^^–^^30^ cortical response to electrical stimulation,^31^ epileptiform activity,^20^^,^^32^^,^^33^ and lesion delineation.^22^^,^^34^^,^^35^ Within a decade and a half, its capacity as a precise functional mapping tool in humans was established. The next, and perhaps the most important, step was to establish its use as a clinical tool to improve patient care.

Despite the number of demonstrated applications, human ISOI failed to spread from the few centers where this method had been pioneered. A number of factors have contributed to this. First, until such technology becomes routine, a strong collaborative effort between clinicians and researchers is needed. This requires both researchers experienced in ISOI and clinicians willing to devote effort to its development in the OR. Second, many of the early experiment designs, which were adapted from animal studies, utilized episodic stimulus presentation designs that required many trials and thus long (>30  min) experiment times, making it less compatible for the human OR. The early studies thus had only a small number of subjects, resulting in insufficient pilot data for clinical trials. Third, partly due to the limited number of patient studies, a standard ISOI system has yet to be specifically designed for clinical use; consequently, optical imaging setups utilized in the laboratory have been adapted for the OR (e.g., *ad hoc* attachment of the ISOI camera to an operative microscope), introducing another source of variability. Fourth, the operative setting poses additional uncontrolled conditions that lead to unfavorable signal to noise ratios. Such conditions include variability in ambient light due to the screens to monitor patient vital signs as well as large patient/camera vibrations that may be caused by the presence of clinical tools such as sequential compressive devices used during operative procedures. Thus, as of today, there is no standard ISOI method available for intraoperative use.

Recently, there has been a resurgence in efforts to improve and standardize the application of ISOI in the OR. Several groups have addressed these limitations by implementing better experimental setups^36^ and new methods of data acquisition and analysis^37^^,^^38^ that shorten the time needed to conduct experiments. Such improvements have led to larger patient studies, and the largest study to date had 41 patients, Sobottka et al.,^39^ and increased confidence in this approach. Moreover, the field of ISOI in animals continues to evolve; its use in conjunction with new focal stimulation methodologies such as optogenetics,^40^ microelectrical stimulation,^41^ and laser stimulation^42^ ushers in a new age of functional tract tracing. Thus, as the field develops, its potential for use in humans also reaches exciting new heights and points toward more widespread clinical and scientific impact.

## 2 Basis of Optical Imaging

### 2.1 Intrinsic Signal

The “intrinsic signal” referred to in this review is the early hemodynamic responses in brain tissue related to neuronal activity.^1^^–^^6^^,^^26^ In brief, these include local changes in the oxy- to deoxy-hemoglobin concentrations as well as a local increase in cerebral blood flow. These hemodynamic responses lead to changes in the absorption spectrum of the surrounding tissue that can be detected by a sensitive camera. These changes in light reflectance are small, on the order of a 1% signal change, with timecourses that peak a few seconds after the onset of neural activity. These signals are thought to correspond to the so-called “initial dip” in fMRI studies and occur prior to the blood oxygenation level-dependent (BOLD) signal. There is typically a several (5 to 10) second refractory period before the reflectance change returns to baseline.

There are many components in tissue that lead to reflectance changes. Different components are better detected at different wavelengths of illuminant. Three of these components are: (1) total hemoglobin (HbT), used to infer cerebral blood volume (CBV), is the dominant source of signal with green-yellow light (∼500 to 599 nm), (2) deoxyhemoglobin (HbR) is the dominant signal source with red light (∼600 to 699 nm), and (3) changes in cellular swelling are the dominant signal source in the near-infrared spectrum (∼700 to 800 nm).^6^ These hemodynamic changes are induced by both neuronal spike activity as well as subthreshhold synaptic activity and represent summed activity of a population of neurons.

### 2.2 Basics of Intrinsic Signal Optical Imaging

In practice, changes in light reflectance are detected by a CCD camera positioned over cerebral cortex. The relative change in reflectance (dR/R) is measured relative to a baseline control level of reflectance. Because the signal size of intrinsic signals is small, the sensitivity of the imaging camera is a critical variable. Current turnkey ISOI systems (e.g., Optical Imaging, Ltd. Imager 3001/S, SciMedia MiCAM02, Redshirt Imaging LLC NeuroPlex system with a 14-bit NeuroCCD-SM256 camera) come with high frame rate, high pixel count, and low noise 12- to 14-bit cameras. Other requirements for ISOI include selecting the camera optics that determines focal depth and the field of view. For human ISOI, the typical optics used are those provided by the operating microscope upon which the camera is attached via a side port. Imaging wise, this approach might not be ideal due to light losses, the increased depth of field, and the relative motion noise due to vibrations in the OR and between the brain and operating microscope. Illumination is typically provided by operating microscope or halogen light source which is subsequently band-pass filtered to select a wavelength to collect the desired hemodynamic responses. As brain motion noise due to pulsation and respiration is large in human ISOI, the brain is stabilized with a sterile glass footplate (but see Ref. 39). To further increase the signal to noise ratio, multiple (e.g., 6 to 20) trial stimulus presentations are collected, image alignment algorithms are used to correct for any apparent brain motion, and for image analysis spatial and/or temporal filtering is used. There currently is no standard human ISOI approach that has been proven to provide the sensitivity observed in animal studies. Recent advances in imaging cameras and analysis methods may make human ISOI more feasible.^43^ Toward that end, Sobottka et al.^39^ imaged the largest sample of patients (N=41) using three different camera setups and two analysis approaches to increase the signal to noise of the ISOI data. By increasing the signal-to-noise ratio, these methods can provide functional maps with spatial and temporal resolution on the order of tens of micrometers and tens of milliseconds, respectively.^1^^–^^6^ As cerebral cortex in animals and in humans is characterized by functional domains a few hundred microns in size, ISOI has been highly instrumental in revealing the characteristic functional organizations of different cortices.

### 2.3 Intrinsic Signal in Human Studies

Toga et al.^44^ published one of the first studies of intraoperative ISOI to look at the temporal and spatial evolution of optical signals. In seven patients undergoing tumor resection, ISOI maps in SI were directly compared with somatosensory evoked potentials (SEPs). Consistent with studies in animals, observable signals appeared within 1 to 2 s, peaked at 3 s, and disappeared by 9 s. In response to local stimulation of skin, these signals colocalized with the largest SEPs in sensorimotor areas and were topographically appropriate. Cannestra et al.^28^ provided a subsequent report on the time course of the signal with similar findings. These studies introduced the feasibility of using ISOI as an intraoperative mapping method.

A further study on the topographic specificity of ISOI underscored the spatiotemporal specificity of the intrinsic signal. In the OR environment, tactile or electrocutaneous stimuli used for mapping are suprathreshhold, meaning they can induce responses not only from the stimulated skin site but can also activate adjacent sites. Consequently, the responsive cortical area can then be larger and appear less topographically specific. Cannestra et al.^27^ examined this question in a study of nine patients where separate ISOI maps were obtained when different skin areas were stimulated individually. Maps of ISOI peak responses showed unique, nonoverlapping activation patterns; whereas areal responses showed regions of overlap. Significant specificity in regard to timing of signal onset was observed during the early phase of the optical response (500 to 1750 ms) while later responses were nonspecific. Areas of peak ISOI signal corresponded to regions identified with SEPs.

The timecourse of the intrinsic signal is also relevant for stimulus timing considerations. Due to the relatively slow timecourse of the hemodynamic responses, on the order of seconds, repetitive and continuous stimulation paradigms can result in reduction and loss of responses to subsequent stimulus presentations. Thus, unlike cortical EPs which have a higher temporal resolution, hemodynamic responses are relatively slow.^30^ Thus, for event related stimulation paradigms, it is better to incorporate sufficient interstimulus intervals (e.g., 6 to 10 s) to permit recovery to baseline prior to the next stimulus presentation.

It is important to note that the temporal and spatial specificity of optical signals differs from that of other functional mapping methods. In one study,^29^ eight patients underwent mapping with fMRI, SEPs, and ISOI. Each modality provided unique spatial and temporal profiles. While SEPs were the most temporally specific, SEPs and ISOI signals were detected with similar spatial distributions. When fMRI and ISOI were compared, in colocalized activation regions, the temporal profile of fMRI showed an initial decrease (the initial dip) consistent with the intrinsic signal.^45^ In a second study,^46^ ISOI and fMRI data were collected in five patients. This study found that in response to somatosensory stimulation, the late positive phase of the optical signal correlated in time and space with the BOLD fMRI signal, though not precisely. The mismatch between BOLD and ISOI mapping was likely largely due to the different vascular sources of the hemodynamic signals. Early ISOI signals derive from microcapillaries and are a consequence of a decrease in oxyhemoglobin after neuronal activation. The BOLD signal, on the other hand, is dominated by large vessels and is related to the local increase in oxyhemoglobin that over-compensates for the initial decrease in blood oxygen. This underscores the greater spatial precision of ISOI compared to fMRI.^3^

## 3 Electrical Stimulation Mapping

**Fig. 1:**
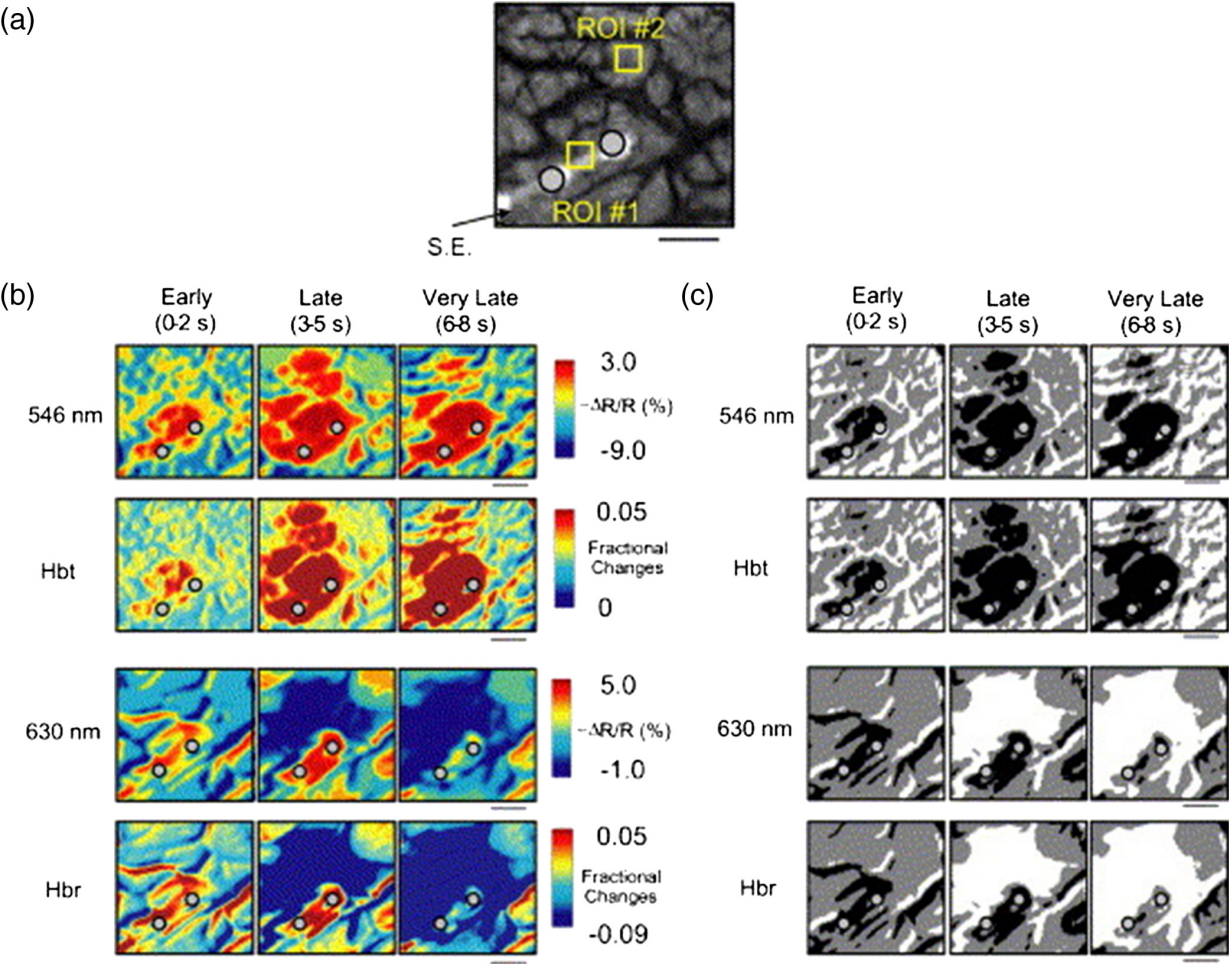
Latency and amplitude dependence of intrinsic optical signals. The “initial dip” in hemoglobin oxygenation occurs earlier and more focally than CBV. (a) Surface of the brain with stimulating electrode (SE) under glass footplate at 546 nm. The recording electrode is outside the field of view. The yellow boxes are ROIs from which the data in Ref. 31 are taken. ROI 1 sits between the SEs, and ROI 2 sits over an adjacent gyrus. Scale bar: 1 cm. (b) Intrinsic signal recorded at 546 and 630 nm at varying latencies after stimulation shows that, although the amplitude of the signal at 546 nm is larger than at 630 nm, the initial changes in signal recorded at 630 nm are more focal than the change in signal recorded at 546 nm, even within the first 2 s after stimulation. Images recorded at 546 and 630 nm were nearly identical with calculated HbT and HbR images (r=0.89, P<0.05). As time passes, the HbT signal spreads diffusely throughout the cortex. At increasing latencies, a decrease in HbR, consistent with the BOLD effect, appears in widespread cortical areas around the SE as well as in the region of the “initial dip.” (c) Statistically significant area of activation recorded at 546 and 630 nm and statistically significant area of calculated HbT and HbR images at different latencies after stimulation. The area of activation was determined based on statistically significant changes in light reflection for each pixel (3 SD above or below the baseline). Example is from a different patient than shown in Fig. 1.^31^ Location of SEs shown with gray circles. Scale bar: 1 cm.^31^ Figure and legend reprinted from Ref. 31, with permission from Elsevier. Imaging methods: an Imager 3001 system (Optical Imaging Ltd.) was used to collect the images. The camera was draped and suspended above the patient with a custom-made camera holder as shown in Ref. 31 (Fig. 1). Light filtered at 546 and 605 nm was provided through a ring illuminator. A sterile glass footplate, placed on the brain, was used to dampen movement artifacts caused by heartbeat and respiration. Strip electrodes placed on the surface of the brain were used for electrical stimulation and recording of surface potentials. Optical recordings at 546 and 630 nm are not pure measures of HbT (CBV) and HbR; HbR reflectance changes at 630 nm can be contaminated by large increases in HbT. Thus, calculations using a modified form of the Beer–Lambert law were used to determine changes in HbT and HbR.

ESM is a procedure that applies electrical stimulation directly to cortex and observes changes in behavior in order to identify areas of eloquent cortex; however, little is known about how ESM disrupts cortical processing or the exact spatial distribution of neurons being stimulated or how it affects the underlying neurovasculature. Reports using ISOI to study ESM have been published. In a key study, Suh et al.^31^ investigated optical signals elicited by bipolar cortical electrical stimulation in eight patients. Figure 1 shows for one patient the hemodynamic response to ESM. They found, in contrast to the small cortical hemodynamic responses generally recorded after sensory stimulation, large changes produced by direct cortical stimulation. Still, HbR was highly localized to the area of neuronal activity, but in addition, increases in HbT were initially equally as focal as HbR and thus could provide another source for high-resolution cortical mapping in humans. Changes in CBV and HbR spread rapidly to surrounding cortical areas and adjacent gyri. These hemodynamic effects were intensity dependent within the range of bipolar stimulus amplitudes (1 to 4 mA) used in the study. With the observation of dramatic changes in the cortical hemodynamic responses, the authors suggested that high amplitude ESM mapping could disrupt cortical activity through a mechanism of transient focal ischemia.

Lavine et al.^38^ investigated ISOI signals elicited by ESM at higher stimulus intensities: 4, 8, and 14 mA in a single patient using light at a wavelength sensitive to HbT (535 nm). They found a high degree of trial-to-trial repeatability in the peak response elicited by ESM. Furthermore, there were either small or no significant differences in the optical responses between awake and anesthetized conditions. Thus, ESM reliably elicits a focal optical response that increases in area and amplitude with increasing stimulus intensity that is relatively immune to anesthesia. These studies provide insight into the spatial distribution and neurovascular changes produced by ESM giving physicians a more complete context to interpret ESM findings. In cases when ESM is required, these findings could be used to develop standard intraoperative stimulation parameters to designate standard areal measurements of activation. This would allow more precise areal delineation with ESM.

## 4 Studies on Human Cortical Function

### 4.1 Somatosensory Cortex

**Fig. 2:**
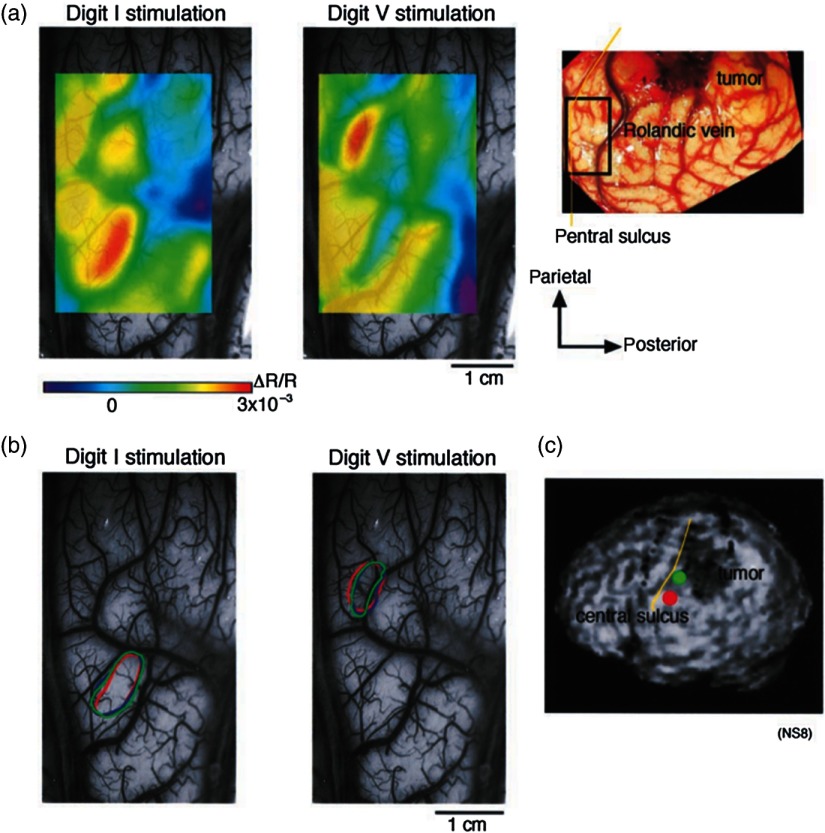
Intrinsic optical responses induced by digit I and V stimulation. (a) Intrinsic optical images recorded from the left SI of a 63-year old patient (case 8 in Table 1 of Ref. 23). Right digits I and V were individually stimulated and the detected optical responses are illustrated by pseudocolor images. The black square in the right panel represents the detected area, and the yellow line indicates the central sulcus. (b) Traces of the optical response areas induced by three repetitive trials. The traces are superimposed for each trial. (c) Equivalent current dipoles (ECDs) are superimposed on a 3-D MR image. The red closed circle is the ECD to digit I stimulation, and the green closed circle is the ECD to digit V stimulation.^23^ Figure and legend reproduced from Ref. 23, with permission from Oxford University Press. Imaging methods: an Imager 2001 system (Optical Imaging Ltd.) was used to collect the images. The camera was fitted onto the operating microscope. Xenon light generated by the surgical microscope illuminated cortex that was filtered at 605 nm. A sterile glass footplate was used minimize brain movement artifacts. Stimulation of digits I and V consisted of 10 mA DC electrocutaneous pulses (n=10) presented at 5 Hz for 2 s.

SI contains a highly complex network of neurons that work together to synthesize information about position, texture, pressure, temperature, etc. of an object in contact with the skin. Precise information about the location of SI and organization allows for its preservation by clinicians and in the future may serve as a blueprint for interfaces integrating sensory information from prosthetic devices. Many of the early studies using ISOI in humans looked at the functional organization of SI with median nerve stimulation, digit stimulation, and trigeminal nerve/facial stimulation. In five patients Sato et al.^23^^,^^24^ and in one patient Shoham et al.^26^ found electrical stimulation of the median nerve resulted in hemodynamic changes in the median nerve territory of SI as confirmed with electrocorticography (ECOG). In a study designed to investigate the specificity and reliability of ISOI, Sato et al.^23^^,^^24^ reported on his observations from stimulating individual fingers. Figure 2 exemplifies these findings by showing the reliability of the response to digit simulation over multiple trials.^23^ Electrocutanous stimulation of digit I and V revealed separate cortical representations along the crown of the postcentral gyrus near the central sulcus. This was one of the first studies to confirm the hand homunculus in humans with functional imaging. Furthermore, in four of the cases, the following pattern was observed: stimulation of D1 and D5 introduced neural responses in two different areas. The first was located near the central sulcus, where D1 and D5 were separately represented. The second response area was near the postcentral sulcus, where D1 and D5 had overlapping representations. In addition to showing that intraoperative, ISOI is a highly effective imaging technique to monitor cortical activity during neurosurgery, with the high spatial resolution of ISOI this was the first study to functionally distinguish Brodmann subdivisions of SI within the same gyrus in humans. In a subsequent study by the group,^24^ stimulation of digits, D1 to D5, was conducted and SI was imaged in six patients yielding confirmatory findings.

Sato et al.^23^^,^^24^ also studied ISOI activation in the facial distribution of SI. In two patients, stimulation of the supraorbital (a branch of V1) and mental (a branch of V3) nerves revealed an activation pattern of overlapping and nonoverlapping areas likely indicating different subdivisions within the facial representation of SI (the nonoverlapping representation occurring in Brodmann area 3b and the overlapping representation occurring in Brodmann area 1). In another patient, Schwartz et al.^25^ identified the face region with ECOG and then imaged this area during cutaneous stimulation. The area of cortical activation during stimulation of the upper face was located medial to that of the lower face. They also found significant overlap (∼30%) between these activation areas.

These findings build on knowledge of SI organization derived from animal studies and human studies using other mapping modalities. For example, recent evidence indicates that human digit representation in SI is similar to the digit representation of nonhuman primates, with each digit having multiple areas of representations; in some areas (e.g., area 3b), representations of different digits are relatively distinct, while in others they appear to have more overlap.^47^

Intraoperative ISOI has the potential to provide clinicians with highly detailed somatotopic maps of S1 that, because they are collected intraoperatively, are not subject to brain shift and other changes to cerebral architecture introduced by the operative environment as opposed to preoperative fMRI. These spatially specific, intraoperative maps could someday serve as the interface for prosthetic devices carrying sensory information to the brain.

### 4.2 Visual Cortex

ISOI has been used extensively to study animal visual cortex. However, as the occipital lobe cortex is rarely exposed during neurosurgical procedures, there are very few studies of ISOI in human visual cortex. Sobottka et al.^48^ optically imaged the occipital lobe while visually stimulating both eyes with strobe-light flashes in one patient. Visual evoked potentials (VEPs) were recorded at four different locations across the occipital lobe for comparison. The locations of ISOI responses were consistent with the electrophysiological VEP findings and corresponded to the anatomical location of visual cortex. This proof of principle report opens up the exciting possibility of mapping cortical columns in human visual cortex and to evaluate whether organizational principles established in studies of visual cortex in animals applies to humans.^16^^,^^49^^,^^50^

### 4.3 Language Cortex

The potential for using ISOI to map language areas in humans is one of its most exciting prospects and has important practical advantages in neurosurgery. Moreover, the greater spatial resolution of ISOI over fMRI mapping potentially offers fundamental insights into the cortical organization underlying speech production and comprehension; these functions are unique to humans and can be directly studied only in humans.

In the first study of language with optical imaging,^20^ areas of activation were observed in the posterior inferior frontal gyrus (IFG) (the region traditionally known as Broca’s area) during a naming exercise in a single patient. Interestingly, these areas were not the same as those found to be essential language sites (sites that caused speech arrest during ESM). On the contrary, in the area that caused speech arrest, optical changes were observed in the opposite direction showing a relative increase in HbR during a naming exercise. This distinction was not observed, in three patients where temporal cortex, an area traditionally referred to as Wernicke’s area, was investigated. Imaging revealed areas of activation in two sites that were found to be essential language sites during ESM with additional areas of activation in three other temporal sites, likely secondary language sites. ESM is limited because it reliably identifies only essential language sites; however, secondary language sites are important in language comprehension and can be identified with ISOI. Extending beyond location of language cortices, Cannestra et al.^21^ studied language cortex with ISOI in 10 patients using both object naming and word discrimination tasks. Depending on the task, different activation patterns were observed. Object naming activated both anterior and posterior regions of the IFG. Auditory responsive naming activated a more posterior language area of the superior temporal gyrus (STG) and word discrimination paradigms activated only the posterior region of the STG. The authors suggest that anterior activations in the IFG reflect semantic processing, while phonological processing is the source of posterior activations. In the STG, they suggest more anterior/superior activations arise from phonological processing and more posterior/inferior activations are the result of semantic processing. Additionally, a case of ISOI of bilingual cortical representations during a visual object naming task was reported.^51^ Imaging revealed areas of activation common to both languages as well as language-specific activation sites in the supramarginal (Spanish) and precentral (English) gyri. The authors concluded there are both distinct and overlapping components in bilingual language representation. In both of these reports, findings from ISOI expanded beyond standard functional mapping to better describe language cortex itself.

Use of ISOI to study language cortex is especially important as its use clinically to delineate eloquent cortex with high resolution would allow for more optimal resection while preserving language function. Compared to fMRI, ISOI has the advantage of being conducted intraoperatively and has been proven useful when used in conjunction with ESM (see Sec. 6). Moreover, its use could provide meaningful new insight into our understanding of how language cortex is organized.

### 4.4 Hemodynamic Oscillatory Activity

In multiple species, including humans, large amplitude ∼0.1  Hz oscillations in cortical hemodynamics known as slow sinusoidal hemodynamic oscillations (SSHOs) have been observed. Rayshubskiy et al.^52^ presented two cases, one in which SSHOs were observed using intraoperative ISOI and another where they were not. In the case where SSHOs were present, they were spatially localized and exhibited wave-like propagation. Moreover, they were localized to specific pial arterioles, but not others, indicating that this was not the result of systemic blood pressure oscillations. For the case where SSHOs were observed, preoperative fMRI data collected 4 days prior to optical imaging data also demonstrated ∼0.1  Hz oscillations in the BOLD signal around the same region. The observation of SSHOs is useful in that it could serve as a biomarker for neurological disease and should be taken into account as a potential confounder for fMRI.

## 5 Epileptiform Activity

Optical imaging has recently been recognized for its potential to characterize, predict, and localize epileptic events. Lenkov et al.^53^ and Patel et al.^54^ have described these capabilities in their recent reviews on the imaging of absence epilepsy and preictal hemodynamic changes, respectively. Notably, the presence of a refractory period with ISOI, as described in the section on the optical signal, could prove advantageous clinically when used to identify the precise location of seizure onset before it spreads. The epileptiform activity detected with standard EEG or ECOG has limited spatial resolution and is often difficult to interpret after a seizure spreads. The peak response with subsequent refractory period identified in ISOI could be used to delineate the initial population of neurons firing with epileptiform activity.

Haglund et al.^20^ imaged stimulation-evoked epileptiform after-discharges in five patients. Optical changes increased in magnitude as the intensity and duration of the after-discharges increased. Interestingly, in areas surrounding the after-discharge activity, optical changes were in the opposite direction, possibly representing an inhibitory surround.^20^ After seeing its utility in studying epileptic activity, the group went on to use ISOI to localize neocortical epileptic foci and found changes in CBV, as opposed to HbR, were superior for localizing such foci as the changes were larger in magnitude and less widespread.^32^ Changes in CBV were highly localized spatially and temporally to areas of firing neurons based on ESM and clinical manifestations of a seizure. In one patient who experienced seizure activity from the mouth/tongue area, ISOI revealed large and highly localized blood volume changes at the site of seizure focus and in the tongue motor area. Given that there is limited accuracy in mapping neocortical epileptogenic tissue by traditional means, the authors advocate this potential clinical use of ISOI.^32^ ECOG, the current gold standard for identifying seizure foci, has the spatial resolution of 1 cm on average, whereas ISOI has the spatial resolution on the order of ∼100  μm. Better delineated identification of epileptic foci would lead to better delineated and smaller resections.

To examine whether hemodynamic changes could predict the onset of the seizures, Zhao et al.^55^ utilized ISOI at two wavelengths, one to measure CBV (570 nm) and the other to measure deoxygenated hemoglobin (HbR) (610 nm). In a single patient with a known epileptic focus, three seizures were imaged. The seizure activity, as demonstrated by ECOG, elicited a focal change in reflectance over the epileptogenic focus that lasted the duration of the seizure. The authors showed that a decrease in CBV precedes the onset of spontaneous seizures. Interestingly, they report that hemodynamic changes preceded the onset of seizures by 15 to 24 s and lasted until 180 to 200 s after the offset. Furthermore, even with a large increase in CBV during the spontaneous focal seizure hemoglobin became deoxygenated. Given the development of “closed loop” treatment paradigms for epilepsy,^56^ knowledge of these distinct hemodynamic changes could direct further treatment innovations. In addition, the authors found that an optical signal resulting from an increase in CBV, although slower to develop, has a larger amplitude and is equally as good as the optical signal derived from HbR at localizing a seizure focus.^55^

**Fig. 3:**
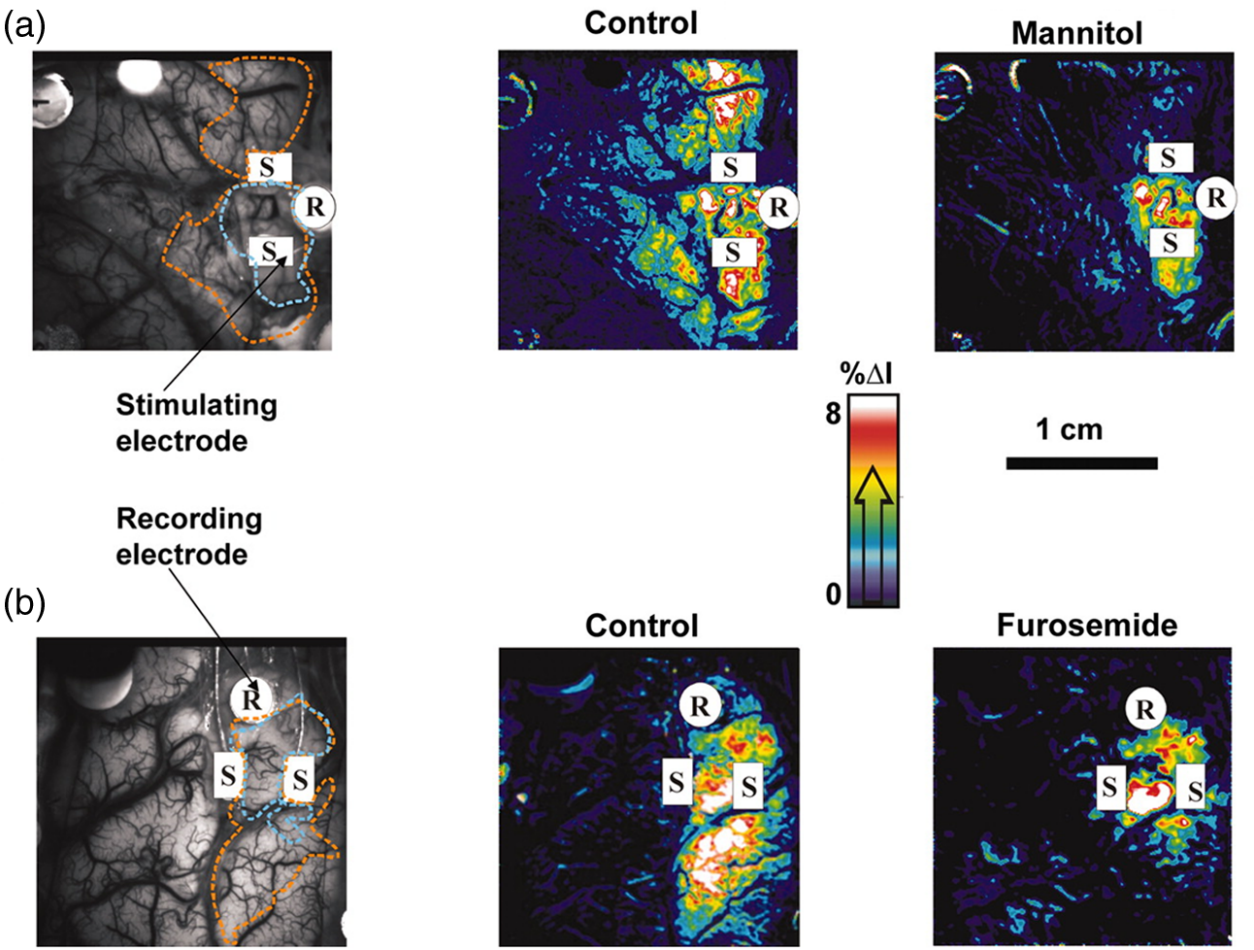
Optical imaging shows reduced cortical spread of stimulation-evoked activity after mannitol and furosemide treatments. Optical imaging was used to map the spatial extent of activated cortex during 60-Hz electrical stimulation. Shown are two different patients, one who was treated with mannitol (a) and another with furosemide (b). The gray-scale images on the left show the appearance of the cortex illuminated with 535 nm (green) light, and the location of the bipolar SEs (marked with “S”) and the recording electrode (marked with “R”). For these studies, the cortices of patients were stimulated for 4 s with current that was 1 mA below the stimulating threshold required for eliciting after-discharge activity. Images acquired at the end of 4 s of subthreshold stimulation were used for comparison. The pretreatment responses of the cortices of two patients are shown in the middle pseudocolored images. Using the same stimulation current, the responses of the cortices were again mapped 30 min after treatment with mannitol (a, right) and furosemide (b, right). Both mannitol and furosemide reduced the spread activation over the cortex by 50% in all subjects. However, the magnitude of the response to electrical stimulation close to the SEs was not reduced. Orange dotted lines on the left gray-scale images show the maximum spread of activity over the cortex before treatments; blue dotted lines show the maximum spread after treatments. Images were pseudocolored to enhance the visibility of small changes; maximum changes (8%) were set to white, and the minimum changes (0%) black.^33^ Figure and legend reproduced from Ref. 33. Imaging methods: a cooled 12-bit digital CCD camera (Roper Scientific, New Jersey) was fitted onto the operating microscope. Cortex was illuminated with 535 nm light provided by four fiber-optic light guides. A sterile glass footplate was used to minimize brain movement artifacts. A bipolar cortical surface electrode provided 4 s of constant current stimulation (a 60-Hz train of 1-ms biphasic pulses) at an amplitude just below after-discharge threshold.

Most antiepileptic drugs focus on altering synaptic transmission; however, modulation of the extracellular space (ECS) may also contribute to epileptogenicity. To address this question with ISOI and direct cortical stimulation, Haglund and Hochman^33^ studied the role of electric field interactions in epileptogenicity by using diuretics to alter the ion concentrations of the ECS in five patients with epilepsy. Figure 3 shows that treatment with intravenous furosemide or mannitol significantly reduced the spread of cortical activation, as monitored with ISOI, evoked by a synchronous train of cortical electrical stimulation. Notably, the magnitude of focal activation at the site of electrical stimulation was not diminished. By suppression of the spread of synchronous stimulation-driven activity, these findings suggest that modulation of the ECS could impart antiepileptic properties without suppressing normal neuronal excitability, an important finding that could direct future pharmacologic therapies. This study points to the potential of ISOI in drug discovery.

Additionally, Hiraishi et al.^57^ used flavoprotein fluorescence imaging (FFI), an ISOI method, to study human cortical specimens from five patients with and five patients without epilepsy. The group found a characteristic cortical propagation pattern that moved horizontally along the cortical layers in specimens from patients with epilepsy and not without. Though this use of ISOI was not conducted intraoperatively, it is an example of ISOI methods being applied for human use with intriguing results.

## 6 Clinical Uses

Within the first decade of ISOI use in humans, its promise as a precise functional mapping tool was established. The next, and perhaps the most important, step to its continued use and development in humans would be to establish its use as a clinical tool that improves patient care. With such sights, Cannestra et al.^22^ developed a system to stratify surgical intervention of perisylvian arteriovenous malformations (AVMs). Twenty patients were stratified into one of three categories based on fMRI of language areas and their relative distance from the AVM. The indeterminate risk group (five patients) underwent an awake craniotomy with ESM and ISOI for language mapping. Three of the five patients were found to have resectable lesions intraoperatively and had no deficits at three months postoperation. The remaining two patients were deemed unresectable at surgery. In these patients, fMRI had shown activation distant from the AVM nidus; however, using data collected from both ESM and ISOI revealed the nidus to be surrounded by areas of language function. Using intraoperative ESM and ISOI mapping allowed thorough mapping of language regions by overcoming the limitations inherent to each technique. The authors advocate use of fMRI, ES, and ISOI to determine language representation before definitive treatment is pursued in patients of indeterminate risk.^22^

ISOI has proven useful as a guide for brain tumor surgery in areas near sensorimotor cortices. Nariari et al.^35^ studied this in 14 patients. To compare ISOI with other functional mapping techniques, SEP and ISOI recordings were conducted in twelve patients; the two modalities provided similar maps in nine of these patients. In the remaining patients, there were discrepancies between the information collected by ESM and ISOI in the region of the central sulcus. Here, the natural bend of the central sulcus or the deformation of the central sulcus caused by mass effect led to the misinterpretation of the phase reversal of the current dipole as measured by SEPs. In addition, ISOI and magnetoencephalography (MEG) were compared in six patients. Once the ECD was projected onto the three-dimensioanl (3-D) brain surface image, the location of the optical signals corresponded with that of the ECD of sensory activation. The authors found that somatotopic information collected with ISOI was useful in determining the resection border in patients with glioma located in the sensorimotor cortex and had superior spatial resolution for delineating the somatotopic representation compared to MEG.^35^

**Fig. 4:**
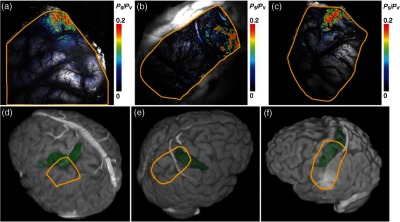
Comparison of intraoperatively generated activation maps with the postoperative anatomical evaluation of the craniotomy site. Cases 25, 33, and 34.^39^ (a)–(c) IOI activation maps after stimulation of the contralateral median nerve. For all three patients, the location of the activated area corresponded well to the intraoperative electrophysiological results (phase reversal in SEP). A similar circumscribed area of activation could be visualized intraoperatively in all patients who had no preoperative sensory deficits. (d)–(f) Exposed cortex area represented in 3-D image space after processing and visualization with Amira software. The yellow line depicts the area of dural opening, green indicates SI. Postoperative evaluation revealed that the location of the activated brain area in IOI corresponded well with the postoperative anatomical evaluation.^39^ Figure and legend reproduced from Ref. 39, with permission from the *Journal of Neurosurgery* Publishing Group. Imaging methods: three different CCD cameras, mounted to an operating microscope, were used: an ORCA-285 (model C4742-96-12G04, Hamamatsu Photonics), an AxioCam MRm (Carl Zeiss MicroImaging GmbH), or an electron bombardment CCD camera (model C7190-13W, Hamamatsu Photonics). Cortex was illuminated with 568-nm filtered light. No footplate was used in this contact free approach. For median nerve stimulation, transcutaneous SEs applied 20 mA of current at a stimulation frequency of 5.1 Hz. Stimulation was presented for 9 min in a block design alternating every 30 s between stimulation and rest periods. After image alignment, to reduce the contribution of noise sources, data analysis was based on Fourier decomposition, an approach that has been successfully used in animal ISOI studies, for instance.^8^ The power spectrum of the timecourse of reflected light of each pixel was computed, where only the spectral energy at the period frequency of the block design (1/60  s) was considered as a stimulus related hemodynamic response. For validation of the ISOI maps, the known phase reversal of the median nerve SEP across the central sulcus and postoperative alignment of anatomical SI landmarks in the 3-D MRIs and position of the craniotomy site (d)–(f) were used. In (a)–(c), $P_{s}/P_{y}$ reports the increase in relative spectral power that would be related to stimulus related hemodynamic response.

These studies, published within a year of each other, seemed to pave the way forward for intraoperative ISOI; however, nearly a decade passed between these and the next study to apply ISOI to clinical decision-making. In a recent report, and for the first time in a large number of patients, Sobottka et al.^39^ evaluated the reliability and validity of intraoperative ISOI. In 41 patients with tumors adjacent to the postcentral gyrus, ISOI of SI was used to visualize activation in response to median nerve stimulation. For validation of the ISOI maps, the known phase reversal of the median nerve SEP across the central sulcus, postoperative alignment of anatomical SI landmarks in the 3-D MRIs, and position of the craniotomy site were used. Figure 4 shows three of the cases where the areas of neuronal activation as measured with ISOI in response to median nerve stimulation were consistent with the postoperative anatomical evaluation. Overall, there was significant (p<0.005), highly reliable differentiation between functional and nonfunctional tissue identified with ISOI; identification of truly functional tissue was 94.4% and the identification of truly nonfunctional tissue was almost 100% when compared with phase reversal and postoperative evaluation. Moreover, the authors found that when using a block versus the typical event related stimulus design used in most animal experiments and data analysis based on Fourier decomposition as a means of removing noise artifacts from the data a high-resolution activity map was available to the surgeon within 12 min. Of note, to acquire the images, no footplate was used, making this ISOI acquisition contact free. Thus, the authors concluded ISOI can be used safely in a routine intraoperative setup and offers the benefit of a high-resolution, highly sensitive, highly specific map of functional activity.

## 7 Challenges

Although ISOI shows much promise for use as a clinical and research tool, some challenges remain. A few of the greatest challenges are related to the size of vascular noise, the time required for image acquisition, motion artifacts, and the lack of standard equipment and procedures for clinical use. A few recent ISOI studies in humans aimed at addressing these challenges have achieved impressive results and provide encouraging indications that such challenges can be resolved.

In standard image acquisition paradigms, the approach for increasing signal-to-noise ratio is to average multiple trials. A few aspects of this approach make it problematic for studies in humans. First, while signal averaging can reduce the relative contribution of noise, in humans’ vascular noise^26^ and cyclical cardiac and respiratory artifacts are quite substantial and can swamp the signal. Second, trial averaging takes time and can prolong surgical time by tens of minutes. As with any intraoperative functional mapping technique, this increased surgical time poses additional risk to the patient. Third, there are quite large cortical motion artifacts in the human which produce poor alignment of image frames, reducing image quality and signal averaging effectiveness. Movement artifacts are typically reduced with the use of a glass plate to stabilize cortex; this glass plate must be placed carefully, as overcompression of the cortex can lead to alteration of physiological signals and potentially ischemic damage.^7^

One potential solution to overcoming these disadvantages is to adopt synchronized signal acquisition approaches. This approach aims to acquire only signals that occur in synch with the frequency of the stimulus presentation; other signals are discarded, thereby removing noise artifacts (such as cyclical noise related heartbeat and respiration) and leaving desired activity-specific signals. Moreover, frame-by-frame alignment procedures^58^ have vastly improved image quality.

In a recent study by Lavine et al.,^38^ dynamic linear modeling methods were applied to data acquired from six human subjects. The results demonstrated significant noise reduction and vastly improved activity-specific mapping. Moreover, maps were acquired in shorter time: imaging series were acquired in 1 to 3 min followed by 15-min analysis, thereby producing images in 18 min. The effectiveness of this approach was also shown in a study by Oelschagel et al.^37^ In this study, a fast Fourier transformation improved signal-to-noise ratio and removed cardiac and respiration noise artifacts. Image displacement due to motion was compensated by using a nonrigid registration method. The application of this method produced reliable images in as little as 12 min. Thus, these novel analysis methods have sufficiently shortened the time needed for acquiring images, making ISOI useful for guiding clinical decisions in the OR and viable for clinical use.

Another challenge is that there is no standard intraoperative ISOI setup. Technical equipment glitches, imaging artifacts, unfavorable operative conditions such as the presence of ambient light, stimulation dependent-complications, depth of anesthesia, and the biological effects of a pathological lesion can all lead to unreliable optical imaging results. Hardware standardized for operative use to minimize equipment issues as well as standardized stimulation paradigms for mapping various cortices (somatosensory, visual, language) and guidelines for anesthesia would all make for improved image quality and ease of use.

To address issues in technical setup, Sobottka et al.^36^ evaluated the clinical practicality of three different intraoperative ISOI camera setups. Interestingly, the group found the image quality of a highly sensitive CCD camera, known as an electron bombarded camera, was up to 10-fold lower compared with two less sensitive cameras. Due to the high sensitivity of the electron bombarded camera, specular reflection (or mirror-like light reflection) and overexposure resulted in poorer quality images. The group went on to develop an ISOI setup using a CCD camera that was less sensitive to aberrant reflections and overexposures, with custom software for intraoperative and postoperative data analysis.^37^^,^^59^ In eight patients, they found that their ISOI setup generated anatomically and electrophysiologically validated ISOI signals of high spatial resolution. They concluded ISOI could be implemented clinically using standard hardware and software standardized for quality and ease of use. It has been reported^39^ that these findings will be supplemented by a prospective German multicenter clinical trial comparing ISOI with standard neurosurgical reference methods and measurements.

We advocate advancement of ISOI as a clinical tool, but when considering ISOI for intraoperative functional brain mapping, its advantages should be compared to ESM, the current gold standard for functional brain mapping. ISOI has advantages to ESM with respect to both patient safety and topographical specificity. Unlike ESM, there is minimal risk of inducing epileptic activity, and it does not necessitate direct contact with tissue (unless there is use of a stabilizing glass plate). Moreover, ISOI has greater spatial resolution and specificity, as current spread during ESM can lead to misinterpretation of essential brain regions.

## 8 Conclusions and Future Directions

ISOI is a well-established imaging modality with exquisite spatial and temporal resolution. In general, it correlates well with ESM, electrophysiological recordings, and fMRI.^34^ In human subjects, ISOI has led to better understanding of the brain. Despite its challenges, it has been successfully used to study human somatosensory,^23^^–^^26^ visual,^48^ and language^20^^,^^21^^,^^51^ cortices, neurovascular dynamics,^27^^–^^30^^,^^44^ and epileptiform activity,^20^^,^^32^^,^^33^^,^^53^^–^^55^^,^^57^ and it shows promise as a clinical tool.^22^^,^^34^^,^^35^^,^^39^

Looking into the future, there are other applications of ISOI that would expand its potential usefulness. Novel hardware and software for two-dimensional optical spectroscopy has been developed that allows recording of optical changes at four wavelengths simultaneously^6^ thus providing information about different elements of cortical hemodynamics. As described above, the Fourier approach, a new method of data collection and analysis, is being introduced to decrease the amount of time necessary to collect the ISOI data.^8^^,^^60^

**Fig. 5:**
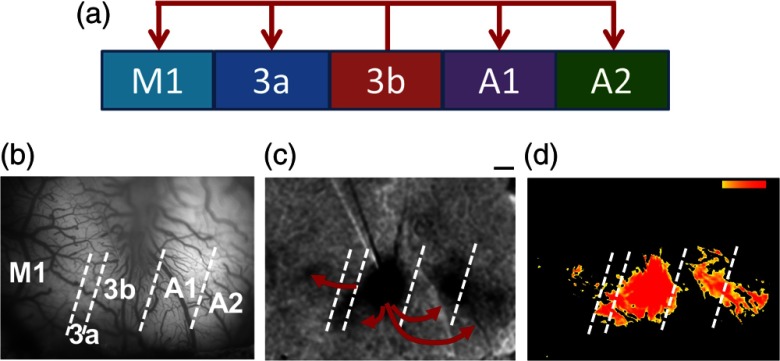
Intracortical microstimulation combined with ISOI reveals focal and distant activation sites. Intracortical microstimulation applied to the digit 4 representation of Brodmann area 3b in an anesthetized squirrel monkey in combination with ISOI revealed a primary activation site at the tip of the microelectrode and additional distant activations in Brodmann areas 1, 2, 3a, and primary motor cortex, a pattern consistent with connections previously identified with anatomical tracing studies.^73^^,^^74^ Methodological details are presented in Ref. 41. ISOI were collected with an Imager 3001 system (Optical Imaging Ltd.) and 632 nm illumination. For the intracortical microstimulation, a 1-MΩ microelectrode was placed in the superficial layers (350  μm) of cortex. Stimulation consisted of a 250-ms duration train of 50-μA biphasic 0.4 ms pulse duration pulses presented at a rate of 250 Hz. (a) Feedforward connections previously identified with anatomical tracing studies.^74^ (b) Vessel map with field of view showing Brodmann areas 1(A1), 2 (A2), 3b, 3a, and primary motor cortex (M1). Dashed lines are approximate borders between Brodmann areas. (c) ISOI map showing a primary activation at the tip of the electrode and additional distant activation sites. Scale bar: 1 mm. (d) A t-map generated with p<0.05 that compared the response between the stimulus condition and the blank (red represents most significant activations). All procedures were conducted in accordance with the National Institutes of Health guidelines and approved by the Vanderbilt University Animal Care and Use Committee and followed the guidelines of the National Institute of Health Guide for the Care and Use of Laboratory Animals.

Other exciting uses of ISOI with potential translational applications in humans are being spearheaded by studies in animals. To name a few, these include using ISOI in animal models of epilepsy,^61^^–^^63^ incorporating ISOI with optogenetic approaches,^64^^–^^66^ and combining ISOI with intracortical microstimulation.^19^^,^^41^^,^^67^^–^^71^ The laboratory of Anna Roe has spearheaded an approach to trace connectivity within and between somatosensory and motor cortices in monkeys with intracortical microstimulation.^19^^,^^41^^,^^72^ Figure 5 shows that when used together with microelectrical stimulation, ISOI can reveal areas of local connectivity around the site of stimulation as well as provide maps of cortical connectivity between areas of the brain at high spatial resolution. In this example in SI of an anesthetized squirrel monkey ISOI revealed activation of cortical circuits around the tip of the stimulating microelectrode and additional distant activations in Brodmann areas 3a, 3b, 1, and 2, and primary motor cortex, a pattern consistent with connections previously identified with anatomical tracing studies.^73^^,^^74^ Use of these novel methods in the OR could reveal specific patterns of connectivity never before observed in humans. As in monkeys, high spatial resolution offered by ISOI would reveal connections between specific cortical columns and lead to understanding of column-based functionally specific networks. As such networks underlie the basis of behavior and cognition, the ISOI approach could also be highly instrumental in understanding network abnormalities in neurological disease.

Noninvasive focal laser stimulation methods are also being developed with an eye toward application in humans^75^^–^^78^ that have the potential of being a less invasive alternative to stimulation with an electrode. Relatively noninvasive focal neural stimulation in combination with ISOI could have profound implications if applied clinically. Imagine prosthetic limbs that could convey the experience of sensation directly to SI. Envision a closed-loop system that could read intention and project sensory input to brain areas allowing for conscious and subconscious corrections to motor function. An individualized highly detailed map of SI could allow for a prosthetic limb that mimics the sensory input and motor output of a true limb. Use of these methods in the OR could reveal patterns of connectivity never before observed in humans that would underlie the implementation of limb specific brain–machine interfaces.
